# Assessment of the vacuolar Na+/H+ antiporter (*NHX1*) transcriptional changes in *Leptochloa fusca *L. in response to salt and cadmium stresses

**Published:** 2015-09

**Authors:** Hamed Adabnejad, Hamid Reza Kavousi, Hadi Hamidi, Iraj Tavassolian

**Affiliations:** 1 Department of Biotechnology, Faculty of Agriculture, Shahid Bahonar University ofKerman, Kerman, Iran; 2 Department of Horticulture, Faculty of Agriculture, Shahid Bahonar University of Kerman, Kerman, Iran

**Keywords:** Kallar grass, Salt stress, Cadmium, NHX1, Semi-quantitative RT-PCR

## Abstract

Sodium/proton exchangers (NHX) are key players in plant responses to salinity and have a central role in establishing ion homeostasis. NHXs can be localized in tonoplast or plasma membranes, where they exchange sodium ions for protons, resulting in the removal of ions from the cytosol into vacuole or extracellular spaces. In the present study, the expression pattern of the gene encoding Na+/H+ antiporter in the vacuolarmembrane (NHX1 gene) in *Leptochloa fusca *(Kallar grass) was measured by a semi- quantitative RT-PCR method under different treatments of NaCl and CdCl2. Results indicated that NaCl positively affected expression levels of *Lf*NHX1, and that the amount of *Lf*NHX1 mRNA increased in conjunction with the rise of salinity pressure, This finding suggests that vacuolar Na+/H+ antiporter might play an important role in the salt tolerance ability of kallar grass. The results also showed that cadmium exposure significantly modulated the mRNA expression of the *Lf*NHX1 gene, suggesting that cadmium exposure disturbed Na+ homeostasis across the tonoplast and decreased the salt tolerance ability of kallar grass.

## INTRODUCTION

Soil salinization has become a pervasive environmental concern and a worldwide agricultural productivity issue. Most crop plants are sensitive to salinity caused by high concentrations of soil salts [1]. More than 800 million ha of land throughout the world are salt-affected, accounting for about 6% of the Earth’s total land area. Out of 1500 million ha of land farmed by dryland agriculture, 32 million ha (2%) are affected by varying degrees of salinity, and out of the current 230 million ha of irrigated land, 45 million ha (20%) are affected by salt [2]. Unfortunately, saline soil is still rapidly expanding because of irrigation with salty water, improper drainage, entry of seawater in coastal areas, and salt accumulation in arid and semiarid regions [3].

Salt stress is mainly caused by sodium ions, whose high concentrations can be toxic to most organisms. Besides ion toxicity, osmotic stress and K+/Na+ ionic imbalances can cause aberrant metabolism such as the inhibition of cytosolic enzyme activities [4].

Plants use two strategies to maintain low Na+ concentrations: sodium exclusion and sodium compartmentation. Plasma membrane-bound Na+/H+ antiporters operate to transport sodium out of the cell [5]. These antiporters are ubiquitous membrane proteins which catalyze the exchange of Na+ for H+ across membranes, thereby playing an important role in cellular pH and Na+ homeostasis throughout the biological kingdom [6].

Salt toxicity can be mainly attributed to Na+-specific damage to the cytoplasm of the cell [7]. Na+/H+ antiporter activities, which are involved in transporting Na+ out of the cytosol into the vacuole and the apoplast, have been detected in both plasma membrane and tonoplast vesicle preparations of different plant species [6]. Physiological studies suggest the role of these Na+/H+ antiporters in plant salt tolerance [8].

The vacuolar Na+/H+ antiporter (NHX1) has long been proposed to play importantroles in salt tolerance. The compartmentalization of Na+ into the vacuole might reduce the deleterious effects of excess Na+ in the cytosol and maintain osmotic balance using Na+ as a cheap osmoregulatory substance, thus enhancing water uptake and salt tolerance of plants [9]. While transporting Na+ into vacuoles, Na+/H+ antiporters use the pH gradient generated by vacuolar H+-translocating enzymes H+ ATPase and PPiase [10].

Blumwald and Poole [11] first reported the existence of Na+/H+ antiporters in tonoplast vesicles of red beet tap roots. Gaxiola et al., [12] found that *At*NHX1, an *Arabidopsis *homologue of the yeast NHX1, was able to suppress some of the phenotypes of the yeast *nhx1 *mutant. They found that *At*NHX1 mRNA level increased in plants treated with 250 mM NaCl or 250 mM KCl for 6 h [12]. Apse et al. [13] demonstrated *At*NHX1 to be localized in tonoplasts, functioning as a Na+/H+ antiporterin *Arabidopsis*. Moreover, overexpressions of *At*NHX1 were shown to improve salt tolerance in both Arabidopsis and tomato plants, further supporting the role of vacuolar Na+/H+ antiporters in salt tolerance [6].

In addition to *Arabidopsis thaliana *[5], vacuolar Na+/H+ antiporter genes have also been identified in various halophytic and salt-tolerant glycophytic species such as *Oryza sativa*, *Atriplex gmelini*, *Suaeda salsa *and *Triticum aestivum *[4, 14-16].

*Leptochloa fusca *L. Kunth, also known as *Diplachne fusca*, is a highly salt tolerant C4 perennial halophytic forage plant that grows well in coastal salt marshes [17]. Due to its characteristics as a typical euhalophyte with both accumulating and excreting properties, it is an attractive model plant for the study of salt tolerance mechanisms [18].

Despite rapid advances in the molecular identification and biochemical characterization of Na+/H+ antiporters in plants, the expression pattern and regulation of vacuolar Na+/H+ antiporter genes have not been vigorously investigated. As mentioned above, the Na+/H+ antiporter has a crucial function in the exchange of Na+ for H+ across membranes, which is important for the plant's salt tolerance due to the fact that it maintains cellular ion homeostases. However, little information is available about the NHX1 gene's response to cadmium stress [19]. Cadmium, an abundant and non- essential heavy metal, is a toxic trace pollutant for humans, animals and plants, added to the environment and transferred to the food chain mainly by industrial processes and phosphate fertilizers [20]. Cadmium can cause many adverse effects on plants including disturbance in metabolism [21], secondary water stress [22] and oxidative stress [23]. Since heavy metals are persistent environmental pollutants, it is necessary to indicate their toxicological effects on immobile organism such as plants [19]. The purpose of this study is to discover gene expression patterns of the *L. fusca *vacuolar Na+/H+ antiporter (NHX1) in response to salt and cadmium stresses.

## MATERIALS AND METHODS

**Plant material and growth conditions: **Seeds of kallar grass (provided by the Department of Agronomy, Ferdowsi University of Mashhad, Iran) were sown in 15-cm pots filled with prewashed sand and irrigated daily with a half-strength Hoagland nutrient solution. Plants were grown in a greenhouse at 26±2 ◦C in a photoperiod of 16 h. Fertilization was performed twice a week with Hoagland solution. NaCl andcadmium treatments were performed on six-week-old seedlings. For the salinity treatment, seedlings were drenched with 150, 300, 450 and 600 mM sodium chloride solutions. For the cadmium treatment, three experimental groups of 50, 100 and 150 µM cadmium chloride were considered. Sampling was performed in a time course of 0, 6,12, 24, and 72 hours after treatment. All treatments started at 8 am to account for the potential diurnal rhythm in the gene expression patterns.

**RNA extraction and cDNA synthesis: **RNAs were extracted from the treated seedlings by Topazol plus Kit (TopazGene, Iran) according to the manufacturer's instructions. Using a RevertAid First Strand cDNA Synthesis Kit (Thermo SCIENTIFIC, # K1691), 1 μg RNA was reversely transcribed to its corresponding cDNA after the DNaseI treatment of the RNA samples. These were later used as templates for the semi-quantitative RT–PCR.

**Semi-quantitative RT–PCR: **To normalize cDNA amounts of each treatment at different time points,the PCR product intensity of actin was considered as the house- keeping gene. Primer pairs for NHX1 (accession number: JF933902) and actin (accession number: FJ603097) are given in Table 1. Each PCR was performed in a total volume of 25 µl containing 10 mM Tris-HCl pH 8.3, 50 mM KCl, 2 mM MgCl2, 200 μM dNTPs (50 µM of each), 0.3 μM of each primer and 1U *Taq *DNA polymerase (Thermo SCIENTIFIC). For amplification, the reaction mixtures were denatured at 94°C for 3 min, followed by 35 cycles at 94°C for 30 sec (denaturation), 37°C for 60 sec (annealing) and 72°C for 60 sec (extension), with an additional extension period of 8 min at 72°C. Amplification was carried out in a thermocycler (Eppendorf, Germany) with a heated lid. PCR reaction products were mixed with a fifth of the volume of gel loading buffer (SinaClone, Iran) and separated by electrophoresis in a 1.5% agarose gel, using a Tris-borate-EDTA (TBE) system (0.5 x TBE = 45 mM Tris-base, 45 mM boric acid, and 1 mM EDTA). Electrophoresis was carried out at room temperature at 75 V for 1.5 h, after which the gels were stained with EB solution (0.015%) in distilled water. The gel images were then visualized with a UV Transilluminator. Experiments were repeated at least twice to verify the results.

**Statistical analysis: **Amplified products were quantified by Total Lab TL120 software (Nonlinear Dynamics Ltd). DNA band intensities amplified from the vacuolar Na+/H+ antiporter gene were normalized compared to the actin gene by dividing the former to the latter. Using SPSS (Version 16.0), a one-way ANOVA was performed followed by Duncan's multiple range test (DMRT) with a critical value of P ≤ 0.01, to analyze statistical differences among the data from different salt and cadmium concentrations at each time point.

**Table 1:** The sequences of primers used to amplify the genes encoding a vacuolar Na+/H+ antiporter(target gene) and actin as reference gene in Semi-Quantitative RT-PCR.

**Genes**	**Primers**	**Sequences (5'-3')**	**Amplicon Size (bp)**
**Vacuolar Na** **+** **/H** **+ ** **antiporter**	NHX1-F	TTCTGGATTGCTCAGTGCTT	200
	NHX1-R	CAGCCAGCATGTAAGAGAGG	
**Actin**	actin-F	CGTACAACTCCATCATGAAG	199
	actin-R	AGTGTTTGGATTGGTGGCTC	

## RESULTS AND DISCUSSION

Previous studies have identified salt tolerance determinants in organisms ranging from cyanobacteria to fungi and from algae to higher plants. However, a complete understanding of these factors in halophytic species requires more investigation. To the best of our knowledge, little information is available regarding salt stress responses of kallar grass at the molecular level. To further examine the key genes that respond to salinity stress and tolerance in this species, the authors of the present study have previously focused on its isolation, characterization and gene expression pattern analyses [24]. In the present study, to characterize the engagement of NHX1 in kallar grass responses to saline conditions, its expression pattern was checked 6, 12, 24 and 72 hours after treatment with 0, 150, 300, 450 and 600 mM NaCl and 0, 50 100 and 150µM cadmium chloride.

**Figure 1:**
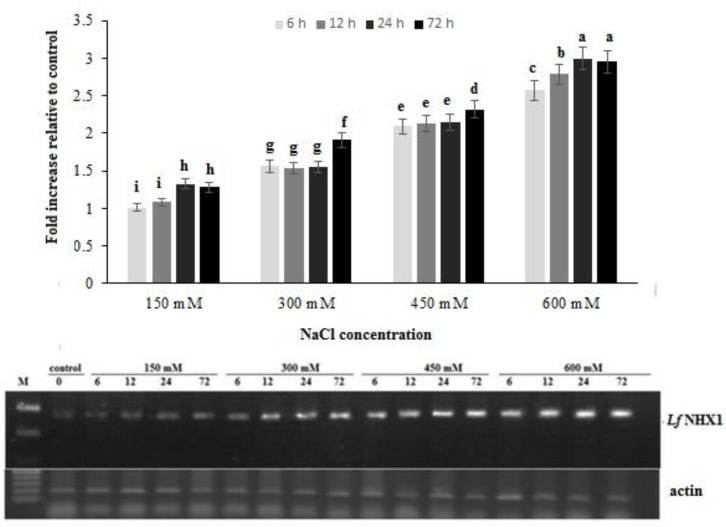
Relative expression levels of *Lf*NHX1 gene after exposure to different concentrations of NaCl. actin gene was used as an internal reference to evaluate the comparative expression level of *Lf*NHX1. Different letters above each column denoted significant difference between them (P ≤ 0.01).

To assess the effect of salt on the expression pattern of the *Lf*NHX1 gene, total RNA from shoots of NaCl treated plants were isolated. Expression levels of *Lf*NHX1 nwere evaluated by semi-quantitative RT-PCR. A basal transcript level was found in non-stressed plants which increased significantly with salt treatments. Quantification of NHX1 transcripts in kallar grass shoot parts showed that salinization affected NHX1 levels positively. In other words, the higher the salinity, the higher the NHX1 levels. In comparison with the control, the relative amounts of mRNA of NHX1 were 1.28, 1.9, 2.3 and 3 fold higher in 150 mM, 300 mM, 450 mM and 600 mM stressed plants respectively after 72 h of exposure to salt (Fig. 1).

NaCl treatment is known to up-regulate vacuolar Na+/H+ antiporter activities and enhance Na+ compartmentation into vacuoles. This up-regulation is either due to the activation of existing proteins and/or the increase of gene transcript levels [24]. Similar results have been reported in *Aeluropus littoralis *[25], *At*NHX1 of *A. thaliana *[26], *Hv*NHX1 of *Hordeum vulgare *[27] *Ag*NHX1 of *Atriplex gmelini *[4], *Ah*NHX1 of *Arachis hypogaea *[28] and *Cm*NHX1 of *Cucumis melo *[29]. In wheat, *Ta*NHX1transcripts also showed increased expression levels in the seedlings after salt treatments [30]. Similarly, the *Sb*NHX1 gene of *Salicornia brachiata *showed very a high expression level with NaCl treatments [31].

Besides its function under salt stress, the role of vacuolar Na+/H+ under normal growth conditions remains to be examined. In the present study, the relatively high basal expression level of V-NHX indicated the important physiological function of *Lf*NHX1 in kallar grass, even in the absence of stress. A shift from purple buds to blue open flowers correlated with the activity of an NHX1 homologue in the Japanese morning glory (*Ipomoea nil*) [32]. A T-DNA insertion mutant of *At*NHX1 showed a much lower Na+/H+ and K+/H+ exchange activity. The mutant plants exhibited altered leaf development and reduced leaf area compared with their wild-type counterparts [33]. These results suggest the Na+/H+ antiporter to be important not only for salt tolerance, but also for the vacuolar pH regulation and the developmental processes of leaves. *Lf*NHX1 was expressed at a high level in the shoots and gradually increased with NaCl stress. After 72 h of exposure to 600 mM salinity, the relative expression of the kallar grass vacuolar Na+/H+ antiporter in the shoots was 3 times larger than that of the controls (Fig. 1). The higher NHX1 expression in the leaves was a prompt response to NaCl treatment which could have helped decrease the Na+ content in the cytoplasm and maintain water concentrations [34]. Such high expression may indicate the sequestration of more Na+ into the vacuoles, hence protecting the cytoplasm against the toxic effects of Na+. This is in line with the fact that Na+ compartmentalization into the vacuoles of leaf cells is an important salt tolerance mechanism [35].

Previous transgenic studies have revealed that the overexpression of the NHX1 gene significantly enhanced plant salt tolerance abilities. In transgenic *Arabidopsis *overexpressing *At*NHX1, higher activities of the vacuolar Na+/H+ antiporter were observed and enabling it to grow in the presence of 200 mM NaCl [13]. The overexpression of *At*NHX1 in tomatos resulted in the growth of transgenic plants,flowers and set fruits at high salt concentrations [36].

The role of sodium compartmentalization in salt tolerance has been further demonstrated in transgenic *Brassica napus*. The transcription level of *Bn*NHX1, a vacuolar Na+/H+ antiporter from *Brassica napus*, increased upon treatment with 200 mM NaCl [37]. The overexpression of the cotton Na+/H+ antiporter gene *Gh*NHX1 in tobacco improved salt tolerance in comparison with wild-type plants [38].

**Figure 2:**
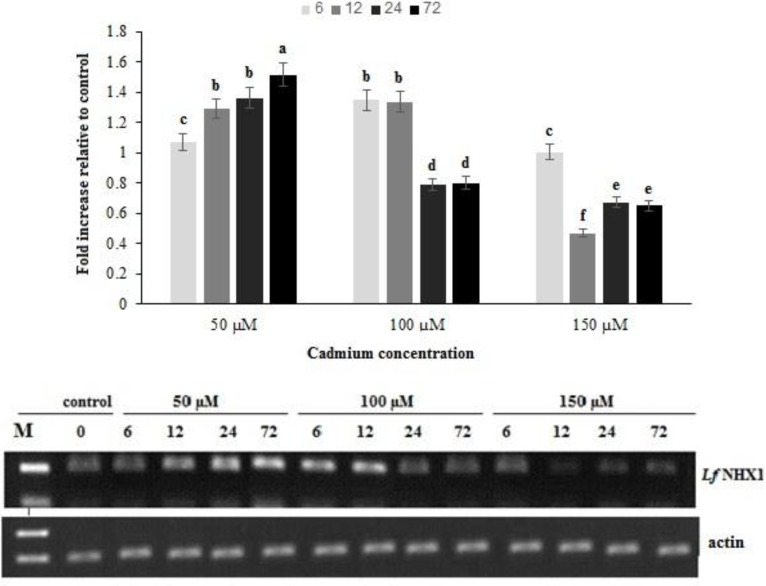
Relative expression levels of *Lf*NHX1 gene after exposure to different concentrations of cadmium. actin gene was used as an internal reference to evaluate the comparative expression level of *Lf*NHX1. Different letters above each column denoted significant difference between them (P ≤ 0.01

The of V-NHX mRNA expression variation profile in response to cadmium is shown in Figure 2, in which no consistent results were discovered with regard to cadmium levels or time durations. In low cadmium concentrations (50 µM group), the the NHX1 gene transcript amount was up-regulated in all sampling times. As time elapsed, increments of the NHX1 gene expression were observed. The increase ofcadmium concentrations in the medium, the transcript levels of the NHX1 gene, gradually decreased and returned to a state which was even lower than basal expression levels of the control group (Fig. 2).

Na+/H+ antiporter is an important membrane protein responsible for pumping Na+ into the vacuole to reduce Na+ toxicity and alleviate the adverse effects of salt stress [38]. In the present study, the investigation of the NHX1 gene mRNA expression in response to cadmium helped understand whether cadmium exposure affected kallar grass salt-tolerance ability. The findings suggest that treatment with 50 µM cadmium enhanced the transcript amount of the NHX1 gene during measured time-courses. Nevertheles, with increasing cadmium concentrations, NHX1gene expression levels and salt tolerance ability decreased in kallar grass.

When cadmium concentration was low in the medium, significant NHX1 transcript enhancements occurred in kallar grass shoots. However, in the medium and high concentration (100 and 150 µM) groups, gene expression levels and salt tolerance ability gradually decreased. This implies that exposure to low cadmium concentrations (50 µM) induces higher salt-tolerance ability in kallar grass. Nevertheless, this effect disappeared with increasing cadmium concentrations and exposure time. Exposure to100 and 150 µM cadmium concentrations impaired the normal homeostasis of Na+ around the membrane and decreased the salt-tolerance ability of kallar grass.

In conclusion, the results suggest that tonoplast *Lf*NHX1 plays important roles in the compartmentation of Na*+ *into vacuoles, and that antiporter activity is probably one of the most important factors determining salt tolerance in kallar grass. Other results showed that kallar grass Na+ homeostasis and salt tolerance ability were also affected by cadmium exposure but exhibited dose-response over time. However, for more insight into the function of this gene further studies will be needed on the interaction between salt and cadmium.

## Notes

**Acknowledgements: **This research was financially supported by Shahid Bahonar

University, Kerman.
